# Performance of Tablet Splitters, Crushers, and Grinders in Relation to Personalised Medication with Tablets

**DOI:** 10.3390/pharmaceutics14020320

**Published:** 2022-01-28

**Authors:** Herman J. Woerdenbag, J. Carolina Visser, Marlyn P. A. M. Leferink op Reinink, Roël R. van Orsoy, Anko C. Eissens, Paul Hagedoorn, Hilda Dijkstra, Derk P. Allersma, Shi W. Ng, Oscar S. N. M. Smeets, Henderik W. Frijlink

**Affiliations:** 1 Department of Pharmaceutical Technology and Biopharmacy, University of Groningen, Antonius Deusinglaan 1, 9713 AV Groningen, The Netherlands; j.c.visser@rug.nl (J.C.V.); m.p.a.m.leferink.op.reinink@student.rug.nl (M.P.A.M.L.o.R.); r.r.van.orsoy@student.rug.nl (R.R.v.O.); a.c.eissens@rug.nl (A.C.E.); p.hagedoorn@rug.nl (P.H.); h.w.frijlink@rug.nl (H.W.F.); 2 Department of Clinical Pharmacy and Pharmacology, University Medical Center Groningen, P.O. Box 30.001, 9700 RB Groningen, The Netherlands; h.dijkstra02@umcg.nl (H.D.); d_allersma@hotmail.com (D.P.A.); 3 Royal Dutch Pharmacists Association (KNMP), Alexanderstraat 11, 2514 JL The Hague, The Netherlands; s.w.ng@knmp.nl (S.W.N.); o.s.n.m.smeets@knmp.nl (O.S.N.M.S.)

**Keywords:** oral solid dosage forms, tablet splitting, tablet crushing, tablet grinding, personalised medicine, swallowing problems, paediatric patients, geriatric patients, nursing home, hospital, individualised preparations

## Abstract

Swallowing problems and the required dose adaptations needed to obtain optimal pharmacotherapy may be a hurdle in the use of tablets in daily clinical practice. Tablet splitting, crushing, or grinding is often applied to personalise medication, especially for the elderly and children. In this study, the performance of different types of (commercially available) devices was studied. Included were splitters, screwcap crushers, manual grinders, and electric grinders. Unscored tablets without active ingredient were prepared, with a diameter of 9 and 13 mm and a hardness of 100–220 N. Tablets were split into two parts and the difference in weight was measured. The time needed to pulverise the tablets (crush time) was recorded. The residue remaining in the device (loss) was measured. The powder was sieved to obtain a particle fraction >600 µm and <600 µm. The median particle size and particle size distribution of the later fraction were determined using laser diffraction analysis. Splitting tablets into two equal parts appeared to be difficult with the devices tested. Most screwcap grinders yielded a coarse powder containing larger chunks. Manual and especially electric grinders produced a finer powder, making it suitable for administration via an enteral feeding tube as well as for use in individualised preparations such as capsules. In conclusion, for domestic and incidental use, a screwcap crusher may provide sufficient size reduction, while for the more demanding regular use in hospitals and nursing residences, a manual or electric grinder is preferred.

## 1. Introduction

The oral route is the most common, convenient, and safe way for drug administration. Oral solid dosage forms are preferred by patients and are stable and broadly available from industrial manufacturers with consistent quality. Dose adjustment is commonly achieved through tablet splitting, whereas crushing or grinding (together referred to as pulverisation) are for different reasons also applied on tablets. Pulverisation may be used to ease swallowing, for dose adaptations (e.g., preparing capsules) or for administration via an enteral feeding tube [1,2,3]. Especially paediatric and geriatric patients may benefit from operations personalising the dose, although it should be realised that this usually falls outside of the terms of the drug’s product license.

Multiple factors need to be taken into consideration when determining the appropriate dose and dosage form for paediatric or geriatric patients as they are highly heterogenous populations [2,4,5]. For children, continuous dose adaptations are needed during their growth, according to the size, weight, age, and physiological condition [6,7,8,9]. In the under-18 population, there is considerable physiological and pharmacokinetic variation, due to differences in bioavailability, volume of distribution, organ maturation, and drug clearance [10]. Off-label use of medicines is common in the paediatric population and may require doses that differ from those of commercially available products [11]. Organ impairment (liver, kidneys) and polypharmacy are relevant determinants for dose adaptation in geriatric patients.

Swallowing problems are common in both patient groups. Only at the age of approximately 6 years a child is able to swallow oral solid medication. Elderly frequently suffer from dysphagia, caused by physiological decline (reduction of muscle control in the mouth and oesophagus area, decreased saliva production, xerostomia) as well as diseases (such as dementia, stroke, Parkinson’s disease, oropharyngeal tumours, thyroid disease and diabetes). Dysphagia may also be drug-induced, e.g., by anticholinergic agents, antibiotics, and antidepressants [12,13,14,15,16].

Oral liquid dosage forms may be an alternative to oral solid medication, but only if a product is commercially available or if a standardised preparation is available, e.g., in a national formulary. Oral liquids are easy to ingest, but stability, dose accuracy, palatability, smell, and taste are well-known thresholds for developing them. Novel solid dosage forms, including multiparticulates, orodispersible dosage forms, and minitablets, are therefore gaining in popularity in the target patient groups [4,17,18].

Tablet splitting or pulverisation prior to administration can be done either by the patient or a caregiver (e.g., a nurse). Various methods are used in daily practice, but their suitability and reliability are generally unknown.

The aim of this study was to investigate the performance of different types of (commercially available) tablet splitters, crushers, and grinders, in relation to their use in the domestic setting as well as in the professional setting of hospitals and nursing residences. The information obtained for our study may help to make choices regarding these devices in daily healthcare practice. In this context, the focus is on overcoming swallowing problems, on dose adaptation, and on the suitability of grinded tablets for the production of individual preparations (e.g., capsules) or for administration via a feeding tube. For the latter, a particle size analysis of the obtained powders was carried out. Costs, ease of operation, and cleaning of the devices were taken into consideration as well.

## 2. Materials and Methods

### 2.1. Materials

Microcrystalline cellulose (Pharmacel 102) was obtained from DFE Pharma (Goch, Germany).

Magnesium stearate was obtained from Sigma Aldrich (Darmstadt, Germany).

Kollicoat^®^ IR (polyvinyl alcohol/polyethylene glycol graft copolymer) was obtained from Sigma Aldrich (Darmstadt, Germany).

Talc was obtained from Bufa (IJsselstein, The Netherlands).

### 2.2. Equipment

Powders were mixed using a Turbula mixer T2C, Will A. Bachofen Maschinenfabrik (Basel, Switzerland).

To determine the compaction force of the tabletting machine in relation to the tablet hardness, a Paul Weber hand tablet press with hydraulic mechanism SPX Stone was used (P.O. Weber Laborpress-Technik, Remshalden, Germany). For the production of tablets, a Hoko Indola eccentric tabletting machine (single-punch) (Hoka KJ, Rijswijk, The Netherlands) was used.

The crushing strength of the tablets was determined using a Dr. Schleuniger 6D hardness tester, Schleuniger Pharmatron, Inc. (Manchester, UK).

Coating was done using a Heidolph REAX 2000 and Minipuls 3 coating machine, Heidolph Instruments (Schwabach, Germany).

The laser diffraction apparatus used for particle size analysis was a HELOS/BF (Sympatec GmbH, Clausthal-Zellerfeld, Germany).

### 2.3. Tablets

The powder mixture for tablets consisted of 150 g Pharmacel 102 and 0.75 g magnesium stearate. The ingredients were mixed in a 2L-container for 5 min using the Turbula mixer at 90 rpm.

The setting of the Hoko tabletting machine to obtain tablets of various hardnesses with fixed weight was determined manually, on the basis of crushing strength measurements and weight determinations of tablets made with the Paul Weber hand tablet press.

Batches of 300–400 flat, unscored tablets were produced having the following specifications.

Diameter 9 mm; weight 250 mg; thickness 2.5 mm; and hardness 100, 150, and 200 N.

Diameter 13 mm; weight 500 mg; thickness 3.0 mm; hardness 100, 130, 180, and 220 N.

Biconvex tablet cores, diameter 9 mm, weight 250 mg, hardness 100 N, were produced for subsequent coating with Kollicoat IR.

Kollicoat IR, 20 g, was dissolved in 100 mL water and stirred at 500 rpm for 24 h. Then, 1 g of talc was added and the mixture was thoroughly stirred. Before the coating was started, the tablets were dried in the rotating coating pan for 30 min (temperature approximately 60 °C). Tablets were spray-coated in a mini-rotating drum at 32 rpm with a spray rate of 2.0 mL/min using a peristaltic pump (Minipuls 3, Viliers le Bel, France) connected to a nozzle with a bore diameter of 1 mm (Schlick 970, Düsen-Schlick, Coburg, Germany). The temperature was maintained at approximately 60 °C with a heat gun. After the coating was applied, the tablets were allowed to dry in the drum for 2 min. The coating thickness was about 5 mg/cm^2^.

### 2.4. Devices and Definitions

An overview of the devices tested is given in Table 1. The categories comprise splitters, screwcap crushers (with and without splitter), manual grinders, and electric grinders.

The difference between crushing and grinding is not clearly defined. We consider grinding as a procedure to obtain the finest powder, while crushing may also yield some larger tablet fragments. While crushing tablets is done to ease swallowing, grinding is also (and mainly) done to enable administration through a feeding tube and for the preparation of capsules. Especially for the latter manipulations the particle size distribution of the powder obtained is relevant.

### 2.5. Tablet Splitting

Tablets were split into two (as equal as possible) parts using the splitters listed in Table 1. Individual tablets were weighed and after splitting the weight of each part was determined. The mean difference (%) of two split parts relative to the calculated weight of a half tablet was determined (*n* = 6 for each tablet type).

### 2.6. Crush Time and Grinding Time

The screwcap crushers and the manual grinders listed in Table 1 were tested for crush time and grinding time, respectively; ease of use; and residue remaining in the device after crushing or grinding. The crush time or grinding time was defined as the time needed to obtain a fine powder (free or nearly free from chunks) for six tablets per tablet type, crushed or grinded one by one. The efficiency of the screwcap crushers was evaluated after 30, 60, 90, and 120 s, and that of manual grinders after 10, 20, 30, 40, 50, 60, and 90 s.

### 2.7. Efficiency of Electric Grinders

With the Severo electric grinder, six tablets of each tablet type were pulverised one by one, applying one pulse of 8 s or five consecutive pulses of 8 s.

With the IKA electric grinder, six tablets of each tablet type were pulverised one by one during 3 min at 25,000 rpm. In addition, 10 tablets of each tablet type were pooled and pulverised during 3 min or 1 min at 25,000 rpm.

### 2.8. Loss after Crushing or Grinding

Tablets were weighed before crushing or grinding, and the powder obtained was weighed after being transferred from the device onto a weighing paper. The difference between the weights was due to powder remaining in the device and dust lost in the environment. The residue (loss) was calculated as a percentage of the intact tablet weight.

### 2.9. Median Particle Size and Particle Size Distribution

To evaluate the particle size of the obtained powders, the applied crush time for screwcap crushers and manual grinders was 1 min for all tablet types and 2 min for selected tablet types. The settings of the electric grinders were as described under Section 2.7. After crushing or grinding the single tablets, the pulverised material was pooled and sieved.

The sieve fraction >600 µm was determined and expressed as percentage of the total powder mass. This larger particle fraction was discarded because it would give a distorted picture of the particle size distribution. In addition, such large particles are unsuitable if the powder is used for filling capsules or for administration via an enteral feeding tube. Of the sieve fraction, <600 µm the median particle size (for all devices) and the particle size distribution (only for the electric grinders) were determined using laser diffraction analysis.

The laser diffraction apparatus was equipped with a 500 mm (R5) lens. The measuring range was from 0.5 to 875 µm. The used laser diffraction calculation mode was based on the Fraunhofer theory. Laser diffraction measurements were done at least in duplicate for each tablet size and hardness, in 1.5 g of a pooled sample. The powders were dispersed into the laser beam with a RODOS system at 3 bar. The start of the measurements was triggered on an optical signal of 0.2% on channel 10, and the measurements were stopped either after the signal decreased to a value lower than 0.2% on the same channel for a period of 1 s, or after 3 s of real measurement time. The results are expressed in percentile values, as d10, d50 (median particle size), and d90, indicating the size below which respectively 10%, 50%, or 90% of all particles were found.

### 2.10. Statistical Analysis

Where applicable, results were statistically analysed using the paired Student’s *t*-test. A *p*-value < 0.05 was considered as statistically significant.

## 3. Results

### 3.1. Tablet Splitters

In Table 2, the results obtained with the tablet splitters are presented.

The Pilomat and the Vitility showed the best performance (in terms of accuracy, reproducibility and precision) among the splitters tested. For most tablet sizes and hardnesses tested, the results obtained with these devices were significantly better than those obtained with the kitchen knife, the Pilltool, and the PillAid. With the Pilltool, larger differences between the two parts were found as tablet hardness increased. The Pilltool splitted the tablets skew. The PillAid has a small knife yielding huge differences in weight between the two parts. The results obtained with the kitchen knife were poor as well. All splitters had a small compartment to store (split) tablets.

### 3.2. Screwcap Crushers

In Table 3, the results obtained with the screwcap tablet crushers are presented.

With a screwcap crusher, one tablet at the time is grinded via hand-twist movements. All screwcap crushers were equipped with a small beaker or cup in which the crushed tablet material was gathered. The Pilltool only cracked tablets without further grinding, hence no figures for this device are included in Table 3. For the other three screwcap crushers, the crush time increased with increasing tablet hardness. In many cases, adequate results were obtained after on1e minute of grinding, which we consider as a reasonable time in terms of effort needed for such an operation. Two minutes grinding yielded better results than 1 min for 100 N tablets. For very hard tablets, the screwcap crushers appeared to be unsuitable. Moreover, the vast majority of the particles in the powder had a size > 600 µm, which may be considered too large for refilling into capsules or for administration via an enteral feeding tube. The median particle size of the small powder fraction < 600 µm was around or just above 100 µm. The use of this type device required quite a lot of manual labour. One device (the Vitility) released some plastic particles upon repetitive use. Neither device was able to adequately pulverize the coated tablets.

### 3.3. Manual Grinders

In Table 4, the results obtained with the manual tablet grinders are presented.

With a manual grinder, one tablet at a time can be pulverised. The grinding time with manual grinders increased with increasing tablet hardness. Powders containing only minor fractions of particles >600 µm were obtained after 10–30 s grinding for tablets with medium hardness. For stronger tablets, the pulverising took slightly longer. The median particle size was around 80–90 µm and did not decrease upon increasing grinding time from 1 to 2 min. Little manual force is required to operate devices in this category and, therefore, these devices are considered as user-friendly. The mortar and the PillDrill yielded the finest powder; this was especially observed for the larger and stronger tablets.

### 3.4. Electric Grinders

In Table 5, the results obtained with the Severo electric grinder are presented.

The Severo was only suitable for grinding single tablets, not for more than one at the same time. This device operated in pulses of 8 s. Five of such consecutive pulses were significantly more effective than one when the particle fraction > 600 µm was considered. Tablet hardness had a large effect on the fraction >600 µm in the powder, but the particle size distribution of the powder fraction <600 µm was not affected by the tablet hardness.

In Table 6 and Table 7, the results obtained with the IKA electric grinder are presented.

Milling single tablets for 3 min (Table 6) resulted in powders with still large fractions of particles with a size > 600 µm.

Comparing the particle fraction > 600 µm in Table 6 and Table 7, it is seen that 3 min grinding with a higher tablet load is most efficient. In fact, the IKA grinder should be considered as unsuitable for pulverising single tablets. It was noticed that a lot of heat was produced as a result of 3 min grinding. Therefore, it was decided to reduce the grinding time to 1 min. The results obtained with 3 and 1 min grinding were comparable.

The IKA grinder is equipped with a disposable attachment in which the knifes are located. Replacing this part for each operation avoids the risk of cross-contamination, but increases costs and produces a significant amount of waste.

### 3.5. Coated Tablets

The coated tablets were generally more difficult to pulverise. The screwcap crushers yielded, at best, tablet fragments with large pieces of coating remaining in the powder (data not quantified). The manual grinders performed reasonably well. The median particle size of the powders was comparable with those obtained with uncoated tablets, but there was a considerable fraction with larger parts consisting of mainly coating fragments (Table 4). The electric grinders gave the best results (see Table 5, Table 6 and Table 7).

## 4. Discussion

Tablet splitting, crushing, and grinding are common practice in daily healthcare to overcome a patient’s swallowing problems that may hamper the oral intake of tablets and to achieve required dose adjustments. A prerequisite for tablet splitting, crushing, and grinding is that it is done in a reliable, reproducible, and qualitatively robust way. Subsequently, the pulverised material can be dispersed in liquid or mixed with soft food functioning as a vehicle to ease swallowing [19].

Before considering such manipulations, one must be assured that they will not compromise the dose accuracy, performance of the dosage form, and bioavailability. There is usually no objection to split or pulverise immediate release tablets, but it should never be done with enteric-coated and controlled release formulations. Unscored tablets, very small tablets, or asymmetrically-shaped tablets should not be split either [20]. It must furthermore be realised that certain drugs may cause irritation to the gut mucosa. Finally, the vehicle chosen to ease swallowing may not always be compatible with the drug substance [20]. Ideally, information on these issues is mentioned in the Summary of Product Characteristics of a tablet, accessible via the website of the European Medicines Agency [21]. However, despite its high relevance, such information is regularly lacking.

Splitting scored tablets can be done manually, by pushing thumbs on each side of the tablet until it breaks, or by pushing a biconvex tablet on a hard surface. However, a score line is a prerequisite. Mechanical splitting with a dedicated device can, in principle, also be done with unscored tablets, as the tablet will be positioned in the device in such a way that the knife cuts in the middle. Splitting tablets should be avoided when active ingredients are very potent and show a narrow therapeutic window or a steep dose-response curve. Skew splitting and unequal splitting, as observed, may be the result of the size of the knife, its placement in the device, and the position of the tablet in the splitter.

The European Pharmacopoeia sets requirements for the compliance with uniformity of mass for subdivision of tablets [22]. The mass of a split part should lie within the range of 85–115% of the tablet mass. This criterion is only met with the Pilomat device for all tablets and for the 13 mm tablets with the Vitility and the Pilltool (except the 220 N 13 mm tablets). All other devices yielded results beyond these limits (see Table 2).

For the loss of mass after splitting, no pharmacopeial requirements exist. It has been proposed that the loss of mass after splitting should be <3% [23]. Loss of mass due to splitting of the tablets in our study was neglectable (data not shown).

Our findings that splitting tablets is often unreliable and does not meet pharmacopeial requirements, are in line with earlier studies [24,25,26,27,28,29,30,31]. Physical characteristics of the tablet, such as the size, crushing strength, shape, mass, and scoring, next to the properties of the device used, are determinants for the splitting performance of a tablet. Our results suggest that the cheapest splitters are the poorest performers. It should be realised that tablet splitting may result in inaccurate dosing [30].

If licenced tablets have a score line, its function can usually be found in the Summary of Product Characteristics (SmPC) as published on the website of the European Medicines Agency (EMA). There are three possible reasons for a manufacturer to use a score line on a tablet: (1) to enable the division of the dose in equal parts; (2) to ease swallowing; (3) only cosmetic, no function. The requirements of the European Pharmacopoeia in the monograph on subdivision of tablets [22] only count for the first category. Splitting tablets without a score line should only be done in case a lower dose is required but unavailable as tablet.

Screwcap crushers (manually operated hand-twist crushers) are in principle suitable for the domestic situation where the aim is easing swallowing. Clear differences were found in performance between the various types tested, but the observed variations in the resulting powder are no problem for the purpose aimed at, as long as the entire dose can be taken by the patient, with or without a swallowing aid. Screwcap crushers appeared to be less suitable for stronger tablets, also in view of the amount of manual labour required.

Manual grinders were effective in a shorter time than screwcap crushers. However, the residue after grinding may be considerable, partly due to electrostatic forces. The plastic bag in which tablets are crushed by the Silent Knight yielded a high loss. Thong et al. reported an average loss of 5.8% for crushed tablets tapped out of crushers. They showed that the loss could be considerably reduced by rinsing the disposable cup or bag with a small volume of water, subsequently to be given to the patient [32]. If the tablet is pulverised in a cup from which the patients can take the medication, there will be no loss by transfer. Nguyen et al. showed significant loss of crushed amiodarone, warfarin, hydrocortisone, and captopril tablets taken up in a predefined volume of water in an oral syringe [33]. Kawakami et al. demonstrated that the addition of dicalcium phosphate or lactose monohydrate to a mortar in which a tablet was pulverised with a pestle, reduced drug loss [34].

Manual grinders require considerably less manual force than screwcap crushers do. The ease of operation and the amount of force needed to use the device should be seen in relation to the setting in which they are used. Manual grinders are therefore more suitable for the professional situation where tablets are pulverised repeatedly and frequently for a number of patients. For occasional use at home, a good screwcap crusher will sufficiently meet the needs, also taking the costs of such a device into account. A prerequisite in the domestic situation is that the patient (or caregiver) should be able to operate the tool. Patients suffering from, for example, rheumatic arthritis, will be unable to do so.

Crushed or ground tablets are easily swallowed when suspended in water or mixed with some soft food [13,35]. When using a vehicle other than water, the compatibility with the active substance should be verified. Dairy products, for instance, interfere with drugs such as tetracyclines and ciprofloxacin [20].

A new development in overcoming dysphagia is the use of oral swallowing gels to ease swallowing of whole, crushed, or pulverised tablets. The gel, a medication accessory, renders a liquid mouthfeel and lubricates the drug product. It shows plastic behaviour, meaning that viscosity becomes reduced upon the application of sheer stress. This facilitates swallowing. Various products with different tastes and compositions are currently on the market, offered by different pharmaceutical companies. Oral swallowing gels should be fully compatible with the medicines taken. This should be verified on the basis of ingredients of the gel and the chemical properties of the drug substance. The gels preferably have an IDDSI (International Dysphagia Diet Standardisation Initiative) thickness (measure for viscosity) of level 4. Lower levels may cause aspiration and a sensation of choking and are considered less safe [36]. As yet, the added value and cost-effectiveness of these products is unclear.

For the future, it would be interesting to investigate the applicability of computational methods (e.g., molecular dynamics) to complement experimental approaches in pharmaceutics such as those describe in the present study [37,38].

Electronic grinders are primarily designated for the professional environment, such as hospitals. Important applications include: (1) pulverising tablets for the production of capsules with a personalised dose and (2) pulverising tablets for drug administration via an enteral feeding tube. A powder mixture for capsules should consist of particles < 180 µm, and the size of all particles should be approximately equal to avoid segregation during preparation. This applies to the tablet powder as well as to other constituents of the capsule formulation [39]. The particle size of powders to be administered via a feeding tube should not exceed 180 µm, to prevent blockage [40]. The electric grinders tested were able to produce powders from tablets that meet the above-mentioned requirements. The homogeneity of the powders, in terms of particle size distribution, was good, while the size of 90% of the particles was <60 µm. With some of the manual grinders, a considerable fraction of particles > 600 µm remained. The classical mortar with pestle and the PillDrill performed best.

When pulverising tablets, the cleaning of the device and the risk of cross-contamination have to be taken into consideration. All screwcap crushers and manual grinders were easy to clean with lukewarm water (on tissue paper) followed by subsequent drying with a soft non-flaking cloth and/or air-drying. Some devices make use of disposable cups in which the powder is deposited. The Severo applies so-called rondels: thin round pieces of plastic that separate the turning crushing pestle from the tablet in the plastic cup. The use of rondels keeps the Severo clean and eliminates cross-contamination risk. After grinding, water or a swallowing gel can be added to the cup, making intake by the patient very easy. One has to be sure, however, that no particles stick to the wall of the cup. The disposable attachments with knives of the IKA mill yield considerable waste. Recycling (with adequate cleaning following a validated cleaning procedure) should be possible but after a certain amount of time, the knives will get blunt.

A final aspect relevant for all medication that is split or pulverised, is health and safety. For the patient receiving the medication, there is a risk of under- or overdosing due to unequal splitting, loss in the grinding or crushing device, or erroneous dose measurement. For some operators in a hospital, the repeated generation of manual force needed to crush hard tablets may be a significant burden. Some strongly acting drugs may harm the operator, the patient, or caregiver, through the inhalation of generated dust containing the drug, or through contact with skin, nose, and eyes [20,41]. Exposure can be limited by using gloves, a mouth mask, or working in a hood or safety cabinet. As risk in this context is the product of intrinsic toxicity of the active substance and exposure to it, especially repeated manipulation must be scrutinized.

Coated tablets, containing strong acting medicines, and substances with carcinogenic, mutagenic and/or reprotoxic properties may only be pulverised under strict controlled conditions, assuring personnel’s safety. These can be provided in a clinical pharmacy department with trained staff and dedicated equipment. For this aim, an electric grinder with a closed and disposable compartment for grinding (such as the IKA) is suitable and will prevent contamination of the environment and operator. Such operations should, however, never be done at a ward alongside the patient’s bed. Penicillin or cephalosporin antibiotics are agents prone to elicit allergic reactions. In case of swallowing problems, antibiotic tablets should be disintegrated in water prior to administration instead of being pulverised.

From a study performed in French hospitals, it became clear that management of drug prescriptions in geriatric patients with swallowing problems was not optimal and may even have iatrogenic effects. Crushing was done with medication for which this manipulation is forbidden, no protective equipment was used, crushing equipment was shared between patients without cleaning, and medication was spilled [42].

In general, there is limited awareness of what is justified and what not, with respect to manipulating oral solid dosage forms to personalise medication or for individual preparations such as capsules. There is a clear role for the pharmacist to educate patients and caregivers, at home, in nursing homes, and in hospitals. This can be done by counselling and/or by giving written instructions (e.g., at wards and at hospital departments). It is also important to involve the patient in the treatment and seek for commitment as this will improve adherence. This can be done by asking the patient about experiences with swallowing oral solids, by discussing possible problems, and by (together) seeking for solutions. The pharmacist is able to perform a risk analysis, to choose tablets that are suitable to be split or crushed, to give information about possible manipulations such as breaking, crushing, and disintegrating in water. The pharmacist is also able to give advice on how crushed or pulverised tablets can be best taken by the patient (whether or not to mix with semi-solid food, or which type of food (e.g., preferably no dairy products). Finally, the patient should receive information about the correct way of taking oral solid medication to cope with swallowing problems. The posture of the head should be upright and ample fluid must be taken [3]. Any modification of a medicine needs to be discussed with the physician who will be advised by the pharmacist.

## 5. Conclusions

Tablet splitting with dedicated devices often gives unsatisfactory results, harbouring the risk of incorrect dosing. The use of a screwcap crusher requires a lot of manual force. These devices are therefore less or not suitable for frequent or repeated use, but useful in the domestic situation. They are not expensive. Manual grinders are user-friendly, effective, and can be applied in the professional setting for occasional to not too frequent use. They are more expensive than screwcap crushers. The electric grinders are most suitable for the professional setting, especially for frequent use. Devices in this category have the highest price.

The purpose of performing the operation should be clear. If a powder is needed for administration via a feeding tube or for capsules as an individual preparation, the particle size distribution is far more relevant than in the case of easing swallowing problems. The screwcap crushers and some of the manual grinders investigated in this study yielded considerable fractions with larger particle sizes, making them only suitable to ease tablet swallowing. For the other purposes, especially the electric grinders are very suitable.

In the professional setting of a hospital or a nursing home, the pharmacists should provide clear and adequate operating instructions (at the pharmacy department and also at the ward). It must be clear which tablets can be pulverised, how and how long with which device, and which tablets may not be crushed (such as enteric-coated and controlled release tablets). The instructions also comprise cleaning, in order to prevent cross-contamination. The role of the pharmacist as an instructor, educator, and quality guard, is unique and indispensable here. In the healthcare chain, the pharmacist is the person par excellence, with profound knowledge of pharmaceutical product care to be used in favour of the pharmacotherapeutic outcome in the patient.

## Figures and Tables

**Table 1:** Overview of devices used for splitting, crushing, and grinding tablets. Type of device, brand name, source from which it can be purchased, and approximate price are given as well as the principle of operation and additional remarks (if applicable).

Type of Device	Name	Source	Approximate Price	Principle and Remarks
Splitter	Kitchen knife, sharp metal blade of 8.5 cm length	Warehouse	€1	
Splitter	Livsane Pilomat tablet splitter	Community pharmacy, internet	€6	Storage function for split tablets.
Screwcap crusher combined with splitter	Livsane Pilltool splitter-crusher-cup	Community pharmacy, internet	€2.80	Storage function for split tablets. Crushing by turning the screwcap. Crushed tablet comes in small cup (for drinking).
Screwcap crusher combined with splitter	PillAid	Community pharmacy, internet	€9	Storage function for split tablets. Crushing by turning the screwcap. Crushed tablet comes in small cup (for drinking).
Screwcap crusher combined with splitter	Vitility	Drug store, internet	€10	Storage function for split tablets. Crushing by turning the screwcap. Crushed tablet comes in small cup (for drinking).
Screwcap crusher	Distinctive	Internet	€20	Crushing by turning the screwcap. Crushed tablet comes in small cup (for drinking).
Manual grinder	Stone mortar and pestle, 10 cm diameter	Haldenwanger	€20	Tablet grinded by turning around the pestle in the mortar.
Manual grinder	Tablet Crusher	Internet	>€100	Top has flat surface with pestle, bottom has holder for cup. Tablet in placed in cup before grinding (turning the pestle).
Manual grinder	PillDrill	Internet	>€100	Metal pestle and metal holder with cup. Tablet is placed in cup before grinding (turning the pestle).
Manual grinder	Silent Knight	Internet	€125	Tablet is crushed in small plastic bag using a lever.
Electric grinder	Severo 3.0 Medicine Grinder	Severo IMS Medical	€350	Table model. Stainless steel grinding head turns around in plastic cup. Rondel separates pestle head from tablet. Suitable for one tablet at a time.
Electric grinder	IKA^®^ Tube Mill 100 control	IKA lab equipment dealer	€1800	Coffee mill principle. Disposable attachment with knives. Suitable for several tablets at a time.

**Table 2:** Performance of tablet splitters. Given is the mean difference (%) of two split parts relative to the calculated weight of a half tablet ± standard deviation (*n* = 6 for each tablet type).

Tablet	Kitchen Knife	Pilomat	Pilltool	PillAid	Vitility
9 mm, 100 N, 250 mg	25.3 ± 10.9	7.0 ± 3.0 *	16.2 ± 8.1	37.5 ± 23.5	12.6 ± 9.6
9 mm, 150 N, 250 mg	30.4 ± 9.7	6.9 ± 3.1 *	19.5 ± 5.7	34.4 ± 7.2	10.6 ± 12.4
9 mm, 200 N, 250 mg	22.2 ± 5.2	5.3 ± 6.0 *	16.4 ± 6.9	38.3 ± 2.0	10.8 ± 4.2
13 mm, 100 N, 500 mg	22.4 ± 11.4	4.7 ± 2.5 *	10.4 ± 0.9 *	26.7 ± 4.1	5.0 ± 3.9 *
13 mm, 130 N, 500 mg	14.8 ± 10.9	5.2 ± 4.0 *	6.8 ± 2.6 *	24.4 ± 3.3	5.4 ± 8.4 *
13 mm, 180 N, 500 mg	22.7 ± 8.1	4.8 ± 3.8 *	9.7 ± 1.5 *	28.1 ± 4.0	4.1 ± 3.8 *
13 mm, 220 N, 500 mg	12.0 ± 6.6	6.3 ± 4.2 *	15.6 ± 2.9	19.9 ± 6.6	5.7 ± 2.8 *

* Compliance with the requirements of the European Pharmacopoeia regarding unit of mass for subdivision of tablets (range 85–115% of the tablet mass).

**Table 3:** Performance of screwcap crushers. Crush time (s) is the time needed to obtain a fine powder evaluated after 30, 60, 90, 120, and 180 s (six tablets, one by one). The percentage loss (mean ± sd, *n* = 6), the percentage particle >600 µm of the pooled samples, and the median particle size of the pooled fraction <600 µm are given after 1 min grinding and, for 100 N tablets only, after 2 min grinding (in brackets).

	PillAid				Vitility				Distinctive			
Tablet	Crush Time (s)	Loss (%) ± sd	Particles >600 µm (%)	Median Particle Size (µm) Fraction <600 µm	Crush Time (s)	Loss (%) ± sd	Particles >600 µm (%)	Median Particle Size (µm) Fraction <600 µm	Crush Time (s)	Loss (%) ± sd	Particles >600 µm (%)	Median Particle Size (µm) Fraction <600 µm
9 mm, 100 N, 250 mg	90	5.5 ± 1.2	94.7 (47.0)	96.5 (91.5)	90	1.8 ± 0.6	70.5 (37.7)	98.4 (100.7)	60	3.2 ± 1.2	100 (6.1)	- (91.9)
9 mm, 150 N, 250 mg	180	6.8 ± 1.2	100	-	90	1.4 ± 0.7	95.2	179.3	90	6.1 ± 1.3	100	-
9 mm, 200 N, 250 mg	No crushing after 180 s				120	1.1 ± 0.4	96.8	120.4	No crushing after 180 s			
13 mm, 100 N, 500 mg	60	3.7 ± 1.1	67.1 (38.6)	96.8	90	0.6 ± 0.5	38.8 (37.7)	93.6	120	2.5 ± 1.0	100 (5.2)	-
13 mm, 130 N, 500 mg	60	6.1 ± 1.5	88.4	102.7	90	2.9 ± 3.9	78.1	118.8	120	1.8 ± 3.3	98.5	168.8
13 mm, 180 N, 500 mg	120	5.2 ± 1.0	97.5	122.2	90	2.3 ± 3.4	86.6	109.6	No crushing after 180 s			
13 mm, 220 N, 500 mg	-	-	99.3	-	90	1.1 ± 0.6	98.9	-	No crushing after 180 s			

**Table 4:** Performance of manual grinders. Grinding time (s) is the time needed to obtain a fine powder, evaluated after 10, 20, 30, 40, 50, 60, and 90 s (six tablets, one by one). The percentage loss (mean ± sd, *n* = 6), the percentage particle >600 µm of the pooled samples, and the median particle size of the pooled fraction <600 µm are given after 1 min grinding and, for 100 N tablets only, after 2 min grinding (in brackets).

	Mortar				Tablet Crusher				PillDrill				Silent Knight			
Tablet	Grinding Time (s)	Loss (%) ± sd	Particles >600 µm (%)	Median Particle Size (µm)	Grinding Time (s)	Loss (%) ± sd	Particles >600 µm (%)	Median Particle Size (µm)	Grinding Time (s)	Loss (%) ± sd	Particles >600 µm (%)	Median Particle Size (µm)	Grinding Time (s)	Loss (%) ± sd	Particles >600 µm (%)	Median Particle Size (µm)
9 mm, 100 N, 250 mg	10–20	14.7 ± 2.4	0	83.9 (79.2)	10	14.5 ± 2.3	3.0	87.5 (85.8)	10	8.0 ± 3.3	1.1	87.1 (79.0)	10	7.9 ± 4.5	1.6	85.3 (88.5)
9 mm, 150 N, 250 mg	30	15.1 ± 3.9	0	83.2	20	11.5 ± 2.8	13.7	93.1	20	9.4 ± 2.8	1.9	83.6	20	7.9 ± 8.1	8.9	87.2
9 mm, 200 N, 250 mg	60	13.3 ± 7.4	0.3	83.3	50	11.7 ± 1.7	16.7	94.5	30	10.3 ± 4.1	8.5	91.7	20	11.7 ± 2.9	13.0	92.3
13 mm, 100 N, 500 mg	10–20	5.0 ± 3.0	0	84.3 (85.0)	10–20	8.8 ± 1.3	2.6	85.3 (81.7)	10	3.8 ± 0.2	0.4	79.8 (75.1)	10	4.8 ± 1.4	5.8	84.3 (83.8)
13 mm, 130 N, 500 mg	30	5.5 ± 1.1	0	91.4	20	8.4 ± 1.3	10.3	88.7	10	9.7 ± 5.5	0.5	75.2	20	9.4 ± 4.5	11.7	86.2
13 mm, 180 N, 500 mg	60	6.5 ± 5.2	0	92.6	30	7.2 ± 3.6	16.4	95.7	20	3.7 ± 1.3	0.7	83.7	20	8.8 ± 2.6	19.0	90.8
13 mm, 220 N, 500 mg	90	6.4 ± 2.4	30.7	85.0	40	8.7 ± 1.3	24.5	98.4	30	2.5 ± 1.5	1.6	87.8	20	6.5 ± 3.3	40.1	93.1
9 mm, coated, 250 mg	30	14.5 ± 10.0 *		77.8	90	7.7 ± 2.4 *		92.7	30	7.1 ± 1.8 *		80.2	30	14.3 ± 5.2 *		87.1

* For the 9-mm-coated tablets, loss included the larger particle fraction, mainly consisting of coating fragments.

**Table 5:** Performance of electric grinder Severo. Percentage loss (mean ± sd, *n* = 6), particle size fraction >600 µm, and particle size distribution of the fraction <600 µm. Grinding time of 1 × 8 s and 5 × 8 s are compared. Six tablets were ground one by one, and the obtained powders were pooled for analysis.

Tablet	Loss (%) ± sd 1 × 8 s	Particles >600 µm (%) 1 × 8 s	Particles >600 µm (%) 5 × 8 s	Particle Size Distributiond 10–d50–d90 (µm) 1 × 8 s	Particle Size Distribution d10–d50–d90 (µm) 5 × 8 s
9 mm, 100 N, 250 mg	4.5 ± 2.0	14.8	4.8	9.6–23.8–57.7	12.2–22.1–47.6
9 mm, 150 N, 250 mg	4.5 ± 2.0	32.3	5.8	9.8–22.8–55.6	13.2–20.0–42.2
9 mm, 200 N, 250 mg	5.7 ± 2.3	63.1	17.1	10.6–25.3–56.7	14.2–23.4–52.1
13 mm, 100 N, 500 mg	2.3 ± 0.7	27.4	8.1	9.2–20.6–49.9	9.6–21.4–46.2
13 mm, 130 N, 500 mg	2.9 ± 0.4	34.9	8.9	10.0–23.0–51.4	9.7–22.3–59.1
13 mm, 180 N, 500 mg	2.6 ± 0.6	60.3	14.7	9.7–21.8–49.4	9.3–19.8–45.4
13 mm, 220 N, 500 mg	2.6 ± 0.9	62.5	14.5	10.0–26.0–78.9	9.0–19.8–45.4
9 mm, coated, 250 mg	3.9 ± 0.6	30.7	13.8	10.2–25.4–74.1	9.7–21.8–45.6

**Table 6:** Performance of electric grinder IKA. Six tablets were ground one by one, for 3 min, at 25,000 rpm and the obtained powders were pooled for analysis. Percentage loss (mean ± sd, *n* = 6), particle size fraction >600 µm, and particle size distribution of the fraction <600 µm.

Tablet	Loss (%) ± sd	Particles >600 µm (%)	Particle Size Distribution d10–d50–d90 (µm)
9 mm, 100 N, 250 mg	16.7 ± 5.0	49.2	14.0–108–318
9 mm, 150 N, 250 mg	24.7 ± 7.1	48.0	46.9–110–310
9 mm, 200 N, 250 mg	12.9 ± 6.3	56.8	43.0–126–466
13 mm, 100 N, 500 mg	11.8 ± 4.2	6.3	10.8–30.5–89.3
13 mm, 130 N, 500 mg	11.5 ± 4.3	41.1	11.2–31.4–123
13 mm, 180 Nm 500 mg	14.5 ± 5.2	11.2	20.5–117–304
13 mm, 220 N, 500 g	9.5 ± 3.4	6.9	19.1–103–392
9 mm, coated, 250 mg	12.8 ± 3.1	40.2	58.6–169–642

**Table 7:** Performance of electric grinder IKA. Of each type, 3 times 10 tablets were ground in one run for either 3 min or 1 min, at 25,000 rpm, and the obtained powders were pooled for particle size analysis. Percentage loss (mean ± sd, *n* = 3), particle size fraction >600 µm, and particle size distribution of the fraction <600 µm.

	3 Min Grinding			1 Min Grinding		
Tablet	Loss (%) ± sd	Particles >600 µm (%)	Particle Size Distribution d10–d50–d90 (µm)	Loss (%) ± sd	Particles >600 µm (%)	Particle Size Distribution d10–d50–d90 (µm)
9 mm, 100 N, 250 mg	3.9 ± 0.4	0.6	9.3–22.6–55.4	2.1 ± 0.2	0.3	9.3–22.6–55.4
9 mm, 150 N, 250 mg	4.6 ± 0.8	0.4	9.2–21.3–48.4	2.0 ± 0.5	2.4	9.1–21.2–48.1
9 mm, 200 N, 250 mg	4.7 ± 0.3	0.2	9.8–21.7–50.3	1.5 ± 0.8	0.5	8.2–18.9–43.2
13 mm, 100 N, 500 mg	2.8 ± 0.3	0.3	7.4–21.0–58.6	0.4 ± 0.3	0.3	7.7–20.2–53.5
13 mm, 130 N, 500 mg	1.5 ± 2.3	0	7.2–21.8–58.8	0.9 ± 0.1	4.0	8.0–19.8–51.6
13 mm, 180 N, 500 mg	1.3 ± 0.3	0	7.3–20.4–53.8	1.0 ± 0.1	0.1	7.3–17.8–43.2
13 mm, 220 N, 500 mg	1.2 ± 0.2	0.4	7.6–19.2–49.1	0.4 ± 0.2	1.1	8.2–20.2–79.2
9 mm, coated, 250 mg	2.7 ± 0.3	0.2	8.5–21.0–53.9	1.6 ± 0.4	0.5	8.7–20.3–47.1

## References

[1] Alderborn G., Frenning G., Taylor K.M.G., Aulton M.E. (2022). Tablets and compaction. Aulton’s Pharmaceutics.

[2] Tuleu C., Orlu M., Wright D., Taylor K.M.G., Aulton M.E. (2022). Design and administration of medicines for paediatric and geriatric patients. Aulton’s Pharmaceutics.

[3] Radhakrishnan C., Sefidani Forough A., Cichero J.A.Y., Smyth H.E., Raidhan A., Nissen L.M., Steadman K.J. (2021). A Difficult pill to swallow: An investigation of the factors associated with medication swallowing difficulties. Patient Prefer. Adherence.

[4] Hanning S.M., Lopez F.L., Wong I.C.K., Ernest T.B., Tuleu C., Orlu Gul M. (2016). Patient centric formulations for paediatrics and geriatrics: Similarities and differences. Int. J. Pharm..

[5] Lau E.T.L., Steadman K.J., Cichero J.A.Y., Nissen L.M. (2018). Dosage form modification and oral drug delivery in older people. Adv. Drug Deliv. Rev..

[6] Ivanovska V., Rademaker C.M.A., Van Dijk L., Mantel-Teeuwisse A.K. (2014). Pediatric drug formulations: A review of challenges and progress. Pediatrics.

[7] Bartelink I.H., Rademaker C.M.A., Schobben A.F.A.M., Van Den Anker J.N. (2006). Guidelines on paediatric dosing on the basis of developmental physiology and pharmacokinetic considerations. Clin. Pharmacokin..

[8] Batchelor H.K., Marriott J.F. (2015). Paediatric pharmacokinetics: Key considerations. Br. J. Clin. Pharmacol..

[9] Lopez F.L., Ernest T.B., Tuleu C., Orlu Gul M. (2015). Formulation approaches to pediatric oral drug delivery: Benefits and limitations of current platforms. Expert Opin. Drug Deliv..

[10] Fernandez E., Perez R., Hernandez A., Tejada P., Arteta M., Ramos J.T. (2011). Factors and mechanisms for pharmacokinetic differences between pediatric population and adults. Pharmaceutics.

[11] Schrier L., Hadjipanayis A., Stiris T., Ross-Russell R.I., Valiulis A., Turner M.A., Zhao W., De Cock P., De Wildt S.N., Allegaert K. (2020). Off-label use of medicines in neonates, infants, children, and adolescents: A joint policy statement by the European Academy of Paediatrics and the European society for Developmental Perinatal and Pediatric Pharmacology. Eur. J. Pediatr..

[12] Stegemann S., Gosch M., Breitkreutz J. (2012). Swallowing dysfunction and dysphagia is an unrecognized challenge for oral drug therapy. Int. J. Pharm..

[13] Schiele J.T., Quinzler R., Klimm H.-D., Pruszydlo M.G., Haefeli W.E. (2013). Difficulties swallowing solid oral dosage forms in a general practice population: Prevalence, causes, and relationship to dosage forms. Eur. J. Clin. Pharmacol..

[14] Drumond N., Stegemann S. (2021). Better medicines for older patients: Considerations between patient characteristics and solid oral dosage form designs to improve swallowing experience. Pharmaceutics.

[15] Mc Gillicuddy A., Crean A.M., Sahm L.J. (2016). Older adults with difficulty swallowing oral medicines: A systematic review of the literature. Eur. J. Clin. Pharmacol..

[16] Barnet N., Parmar P. (2016). How to tailor medication formulations for patients with dysphagia. Pharm. J..

[17] Thabet Y., Klingmann V., Breitkreutz J. (2018). Drug formulations: Standards and novel strategies for drug administration in paediatrics. J. Clin. Pharmacol..

[18] Mitra B., Chang J., Wu S.J., Wolfe C.N., Ternik R.L., Gunter T.Z., Victor M.C. (2017). Feasibility of mini-tablets as a flexible drug delivery tool. Int. J. Pharm..

[19] Breitkreutz J., Boos J. (2007). Paediatric and geriatric drug delivery. Exp. Opin. Drug Deliv..

[20] Paparella S. (2010). Identified safety risks with splitting and crushing oral medications. J. Emerg. Nurs..

[21] European Medicines Agency https://www.ema.europa.eu/en.

[22] EDQM Council of Europe (2021). European Pharmacopoeia Supplement 10.5.

[23] Green G., Berg C., Polli J.E., Barends D.M. (2009). Pharmacopeial standards for the subdivision characteristics of scored tablets. Pharmacop. Forum.

[24] Van Santen E., Barends D.M., Frijlink H.W. (2002). Breaking of scored tablets: A review. Eur. J. Pharm. Biopharm..

[25] Vaes L.P.J., Frijlink H.W., Barends D.M. (2002). The breaking of scored tablets prior to the Ph. Eur. test. Pharmeuropa.

[26] Van der Steen J.C., Frijlink H.W., Rodenhuis N., Barends D.M. (2004). The Ph. Eur. requirement for scored tablets: Sampling procedure and test. Pharmeuropa.

[27] Barends D.M., De Groot D.W., Frijlink H.W., Rodenhuis N., Van der Steen J.C. (2005). Development of an in vivo test procedure for the easy of breaking of scored tablets. Pharmeuropa Sci. Notes.

[28] Barends D.M., De Groot D.W., Van der Steen J.C., De Kaste D., Frijlink H.W. (2006). Results of a market surveillance study in the Netherlands on break-mark tablets. Pharmeuropa Sci. Notes.

[29] Van der Steen J.C., Frijlink H.W., Maarten C., Schipper A., Barends D.M. (2010). Prediction of the ease of subdivision of scored tablets from their physical parameters. AAPS PharmSciTech.

[30] Van Riet-Nales D.A., Doeve M.E., Nicia A.E., Teerenstra S., Notenboom K., Hekster Y.A., Van den Bemt B.J.F. (2014). The accuracy, precision and sustainability of different techniques for tablet subdivision: Breaking by hand and the use of tablet splitters or a kitchen knife. Int. J. Pharm..

[31] Van Gosliga F., Burgler L., Heijnen M., Last T., Van Zanten E., Yska J.P. (2019). Can tablets be split in half manually in a reliable manner?. Ned. Platf. Farm. Onderz..

[32] Thong M.Y., Manrique Y.J., Steadman K.J. (2018). Drug loss while crushing tablets: Comparison of 24 tablet crushing devices. PLoS ONE.

[33] Nguyen D., Secretan P.H., Auvity S., Vidal F., Postaire M., Cisternino S., Schlatter J. (2020). Assessment of practices for suspended oral drugs by tablet crushing in pediatric units. Eur. J. Pharm. Biopharm..

[34] Kawakami M., Kitada R., Kurita T., Tokumura T. (2017). A method for decreasing the amount of the drug remaining on the surfaces of the mortar and pestle after grinding small amount of tablets. Yakugaku Zasshi.

[35] Forough A.S., Lau E.T.L., Steadman K.J., Cichero J.A.Y., Kyle G.J., Serrano Santos J.M., Nissen L.M. (2018). A spoonful of sugar helps the medicine go down? A review of strategies for making pills easier to swallow. Patient Prefer. Adherence.

[36] Malouh M.A., Cichero J.A.Y., Manrique Y.J., Crino L., Lau E.T.L., Nissen L.M., Steadman K. (2020). Are medication swallowing lubricants suitable for use in dysphagia? Consistency, viscosity, texture, and application of the international dysphagia diet standardization initiative (IDDSI) framework. Pharmaceutics.

[37] De Vivo M., Masetti M., Bottgoni G., Cavalli A. (2016). Role of molecular dynamics and related methods in drug discovery. J. Med. Chem..

[38] Allec S.I., Sun Y., Sun J., Chang C.A., Wong B.M. (2019). Heterogenous CPU+GPU-enabled simulations for DFTB molecular dynamics of large chemical and biological systems. J. Chem. Theory Comput..

[39] Woerdenbag H.J., Sznitowska M., Boer-Bouwman Y., Bouwman-Boer Y., Fenton-May V., Le Brun P. (2015). Basic operations. Practical Pharmaceutics.

[40] Lein A., Ng S.W., Bouwman-Boer Y., Fenton-May V., Le Brun P. (2015). Oral liquids. Practical Pharmaceutics.

[41] Logrippo S., Ricci G., Sestili M., Cespi M., Ferrara L., Palmieri G., Ganzetti R., Bonacucina G., Blasi P. (2017). Oral drug therapy in elderly with dysphagia: Between a rock and a hard place!. Clin. Intervent. Aging.

[42] Fodil M., Nghiem D., Colas M., Bourry S., Poisson-Salomon A., Rezigue H., Trivalle C. (2017). Assessment of clinical practices for crushing medication in geriatric units. J. Nutrit. Health Aging.

